# Isolation and Characterization of a Yellow Xanthophyll Pigment-Producing Marine Bacterium, *Erythrobacter* sp. SDW2 Strain, in Coastal Seawater

**DOI:** 10.3390/md20010073

**Published:** 2022-01-14

**Authors:** Sun Wook Jeong, Jung Eun Yang, Yong Jun Choi

**Affiliations:** 1 School of Environmental Engineering, University of Seoul, Seoul 02504, Korea; jeongsunwook@gmail.com; 2 World Institute ok Kimchi, Gwangju 61775, Korea

**Keywords:** xanthophylls, yellow pigment, marine microorganism, *Erythrobacter*, antioxidant

## Abstract

Xanthophylls, a yellow pigment belonging to the carotenoid family, have attracted much attention for industrial applications due to their versatile nature. We report the isolation of a homo xanthophyll pigment-producing marine bacterium, identified as the *Erythrobacter* sp. SDW2 strain, from coastal seawater. The isolated *Erythrobacter* sp. SDW2 strain can produce 263 ± 12.9 mg/L (89.7 ± 5.4 mg/g dry cell weight) of yellow xanthophyll pigment from 5 g/L of glucose. Moreover, the xanthophyll pigment produced by the SDW2 strain exhibits remarkable antioxidative activities, confirmed by the DPPH (73.4 ± 1.4%) and ABTS (84.9 ± 0.7%) assays. These results suggest that the yellow xanthophyll pigment-producing *Erythrobacter* sp. SDW2 strain could be a promising industrial microorganism for producing marine-derived bioactive compounds with potential for foods, cosmetics, and pharmaceuticals.

## 1. Introduction

Carotenoids are lipophilic isoprenoid compounds that widely occur in nature [1]. They are mostly found in living organisms, such as plants, fungi, algae, and bacteria, and play important biological roles in preventing cancer, cardiovascular diseases, and degenerative diseases through scavenging reactive oxygen species [2,3]. Thus far, more than 700 carotenoids have been identified in nature, among which beta-carotene, lycopene, astaxanthin, lutein, and zeaxanthin are widely used as nutritional supplements, cosmetic ingredients, and pharmaceuticals [4]. Natural carotenoids have increasingly been in demand in recent years due to their unprocessed and natural properties compared to chemically synthetic carotenoids, which account for more than 70% to 80% of the total carotenoid market [5]. Accordingly, the discovery of microorganisms capable of producing natural carotenoids and the development of improved industrial microorganisms using metabolic engineering have been attracting attention [6,7,8].

Over the past few decades, various unique xanthophylls, a type of yellow pigment, have been discovered in marine organisms [9]. One of the well-known marine xanthophylls, fucoxanthin, has been found in marine microalgae, such as *Laminaria japonica, Eisenia bicyclis, and Phaeodactylum tricornutum* [10,11,12]. In addition, sulfate-containing xanthophylls, such as nostoxanthin sulfate, zeaxanthin sulfate, and caloxanthin sulfate, were also identified in the *Erythrobacter flavus* KJ5 strain, isolated from a hard coral, *Acropora nasuta* [13]. These marine-derived xanthophyll pigments have provided a clue regarding marine organisms’ ability to survive in harsh marine conditions, such as low temperatures, high salt concentrations, and strong radiation, and most have superior physiological activity compared to land-derived xanthophyll pigments [14,15].

For example, two rare xanthophylls, saproxanthin and myxol, identified from the novel marine bacterial strains *Flavobacteriaceae* sp. o4OKA-13-27 and YM6-073, exhibited over 2.2-fold greater inhibition activity for lipid oxidation than those of plant-derived zeaxanthin [16]. Deinoxanthin, a novel xanthophyll pigment found in the marine bacterium *Deinococcus* sp. AJ005 strain, exhibited more than two-fold higher reactive oxygen species scavenging activity than plant-derived carotenes and xanthophyll pigments such as beta-carotene, lycopene, and astaxanthin [17,18].

These observations inspired us to isolate new xanthophyll pigment-producing marine bacteria. We report the isolation and characterization of a novel marine bacterium identified as an *Erythrobacter* sp. SDW2 strain capable of producing a yellow xanthophyll pigment. In addition, we also confirm that the xanthophyll pigments derived from the SDW2 strain have superior free radical scavenging activity compared to land-derived xanthophyll pigments and carotenoids.

## 2. Results and Discussion

### 2.1. Isolation and Characterization of a Yellow-Pigmented Marine Bacterium

**Figure 1:**
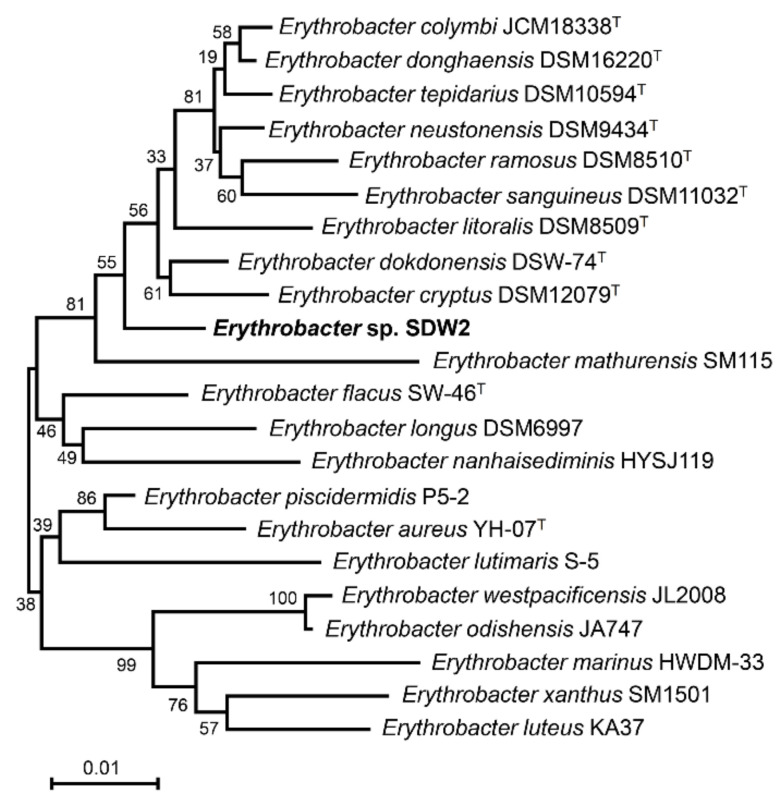
Phylogenetic tree derived from 16S rRNA sequences of *Erythrobacter* sp. strain SDW2 (bold characters) and other closely related bacterial species. Numbers at the nodes indicate the bootstrap value (expressed as percentages of 1000 replications). The superscript ^T^ indicates the type of strain of a species. The bar shows 0.01 substitutions per nucleotide position.

First, a yellow-pigmented marine bacterium designated as strain SDW2 was isolated from coastal seawater in Jeju Island, Republic of Korea. This rod-shaped bacterium was Gram-negative, motile, and aerobic and optimally grew at pH 7 and 37 °C in 2% NaCl. The biochemical characteristics investigated using the Analytical Profile Index (API) test indicated that the bacterium was positive for catalase, oxidase, beta-glucosidase, beta-galactosidase, and nitrate reduction but negative for urease, arginine dihydrolase, protease, and indole production. The bacterium could also use D-glucose, L-arabinose, D-mannose, D-mannitol, D-maltose, potassium gluconate, adipic acid, and maleic acid as a carbon source (Table 1). The phylogenetic analysis based on 16S rRNA sequences with other *Erythrobacter* species revealed that the SDW2 strain was clustered with the *Erythrobacter* genus and exhibited the highest similarity to *Erythrobacter dokdonensis* DSW-74^T^ (98.2%, accession number LZYB01000003; Figure 1). Based on these observations, the newly isolated marine bacterium in this study was identified as *Erythrobacter* sp. strain SDW2.

### 2.2. Characterization of an SDW2-Derived Xanthophyll Pigment

The yellow xanthophyll pigment produced by the strain SDW2 was characterized using the ultraviolet–visible (UV–vis) absorption spectrum, high-performance liquid chromatography (HPLC), and thin-layer chromatography (TLC). The characterization was conducted using crude methanolic extracts obtained from the strain SDW2 when cell growth reached the stationary phase.

**Figure 2:**
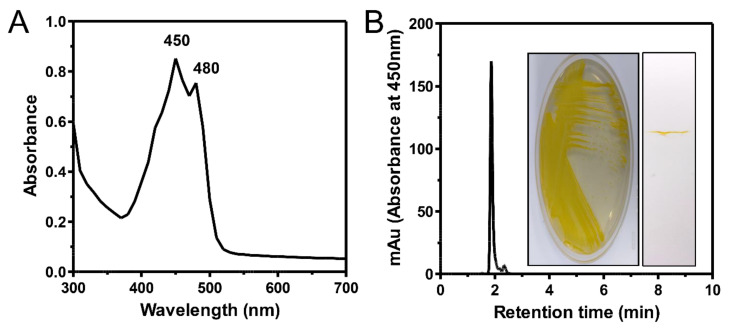
Profiles of total pigments extracted from the *Erythrobacter* sp. strain SDW2. (**A**) Ultraviolet–visible absorption spectrum in the wavelength range of 300 to 700 nm. (**B**) High-performance liquid chromatography (HPLC) analysis of the total pigment extracts at 450 nm with a 1 mL/min flow rate. The insets indicate a purely cultivated SDW2 strain on a marine broth agar plate and an image of thin-layer chromatographic analysis using pigment extracts (50 mg/L).

The UV–vis absorption peak exhibited typical spectral characteristics of yellow xanthophylls (λ_max_ at 450 and 480 nm), such as beta-cryptoxanthin, zeaxanthin, and nostoxanthin, which are produced by some marine bacterial species (Figure 2A) [13,19,20]. In the liquid chromatographic profiles, the total xanthophyll pigment extracts were eluted at a retention time of approximately 1.9 min with 95.2% purity (%area), and a minor peak (4.6% area) was detected at 2.3 min (Figure 2B). Previous reports have shown that *Erythrobacter* species such as *E. flavus* KJ5, *E. longus*, and *E. nanhaesediminis* produce different types of xanthophylls, whereas the SDW2 strain mainly produces only one type of xanthophyll (Figure 2B, inset) [13,21]. This feature can be a notable advantage in the industrial application of microbial-derived pigments as additional purification steps such as saponification and column-based chromatographic separation are not required to recover pure pigments [22,23].

**Figure 3:**
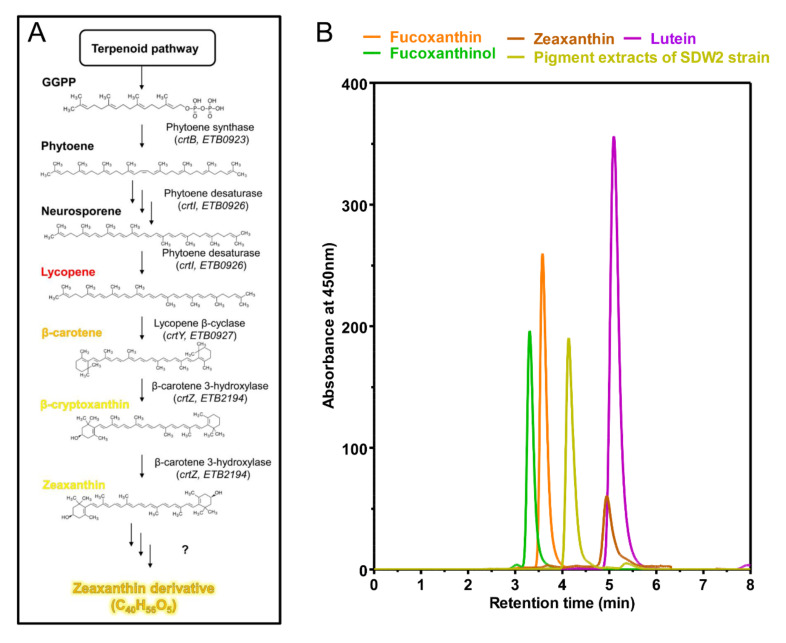
(**A**) Proposed biosynthetic pathway for zeaxanthin derivative in *Erythrobacter* sp. SDW2. (**B**) Profile of HPLC chromatograms using authentic standards (fucoxanthin, fucoxanthinol, zeaxanthin, and lutein) and carotenoid extracts of the SDW2 strain. Xanthophyll biosynthetic pathway was depicted based on analysis of genome sequence of SDW2 strain. ETB numbers given in Figure 3A indicate locus tags of *Erythrobacter* sp. SDW2 genome. GGPP: geranylgeranyl diphosphate. The concentration of standard xanthophylls was adjusted to 20 mg/L. The flow rate was 0.5 mL/min.

To date, the structural analysis of xanthophylls, carotenoids with oxygen functional groups, has been a difficult challenge due to the low oxidative stability and the number of oxygen functions that can generate hundreds of combinations [24]. Thus, whole-genome sequencing analysis was preferred to predict the biosynthetic pathway of xanthophylls and the types of xanthophylls that can be synthesized. Genome annotation analysis of the SDW2 strain revealed that the SDW2 strain has a zeaxanthin biosynthetic pathway composed of phytoene synthase (encoded by the *crtB*), phytoene desaturase (encoded by the *crtI*), lycopene β-cyclase (encoded by *crtY*), and β-carotene 3-hydroxylase (encoded by the *crtZ*) (Figure 3A). Despite having a zeaxanthin biosynthetic pathway, the SDW2-derived xanthophyll pigments were found to be different from those of zeaxanthin and marine microorganism-derived xanthophyll pigments (Figure 3B). Based on these results, the xanthophyll pigment produced by the SDW2 strain was predicted to be a zeaxanthin derivative that can be produced by an unknown metabolic route [25]. Interestingly, highly conserved β-carotene ketolase (encoded by the *crtW*), which is responsible for the conversion of zeaxanthin into other xanthophylls, such as astaxanthin, fucoxanthin, and adonixanthin in *Erythrobacter* species, was found to be absent in the SDW2 strain [26]. These unique metabolic characteristics of the SDW2 strain are superior to other marine bacteria in the mass production of the desired target xanthophyll pigment.

### 2.3. Optimization of Carbon Source for Efficient Production of a Yellow Xanthophyll Pigment

**Figure 4:**
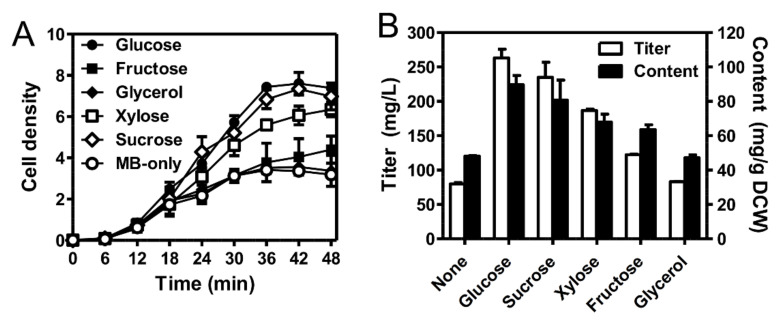
Effect of five carbon sources (5 g/L) on (**A**) cell density and (**B**) xanthophyll pigment production of the *Erythrobacter* sp. strain SDW2 during 48 h of cultivation.

To investigate the production performance of the newly isolated SDW2 strain on the yellow xanthophyll pigment, we tested five carbon sources under pH 7 and 37 °C in 2% NaCl conditions. As shown in Figure 4A, the highest cell growth (OD_600_ of 7.4 ± 0.5) was observed when the SDW2 strain was cultured in marine broth (MB) medium containing 5 g/L of glucose, which was approximately 2.3 times higher than that observed in MB medium without glucose (OD_600_ of 3.2 ± 0.2). The SDW2 strain also grew under conditions in MB medium containing 5 g/L of sucrose (OD_600_ of 7 ± 0.7) or xylose (OD_600_ of 6.4 ± 0.4) as a carbon source but was relatively lower than that obtained in MB medium containing glucose. Furthermore, the SDW2 strain hardly grew in MB medium containing fructose or glycerol. Finally, the SDW2 strain produced 263 ± 12.9 mg/L of a yellow xanthophyll pigment with a content of 89.7 ± 5.4 mg pigment/g dry cell weight (DCW), which is a 69.7% and 46.3% higher concentration and content, respectively, than that obtained using the basal medium (79.8 ± 2.1 mg/L and 48.1 ± 0.3 mg/g DCW) (Figure 4B). As a result, glucose was found to be the best for the production of a yellow xanthophyll pigment among the tested carbon sources. To the best of our knowledge, the xanthophyll pigment production performance of the SDW2 strain is superior to that of other marine microorganisms without additional genetic engineering and process optimization. For example, the newly isolated marine bacterium *Formosa* sp. KMW strain produced 0.69 mg/L of zeaxanthin in shake flask cultivation [27]. In another study, the microalgae *Phaeodactylum triconutum* was cultured for 6 days to achieve the highest fucoxanthin production of 16.33 mg/g DCW [11].

### 2.4. Evaluation of Antioxidative Activity of a Yellow Xanthophyll Pigment

**Figure 5:**
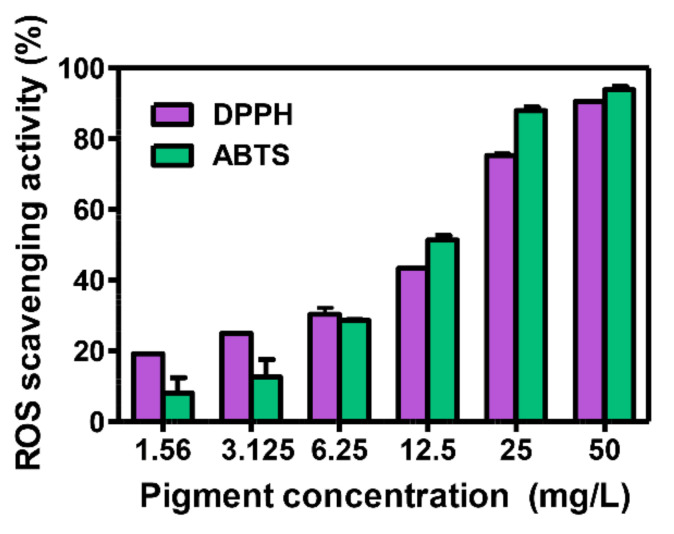
Free radical scavenging activity using various concentrations of the yellow xanthophyll pigment of the SDW2 strain for 2,2-diphenyl-1-picrylhydrazyl (DPPH) and 2,2′-azino-bis(3-ethylbenzothiazoline-6-sulfonic acid) (ABTS) radicals.

Next, the possible use of the xanthophyll pigment extracted by the SDW2 strain as a food additive and pharmaceutical was further explored by investigating the antioxidant activity. To investigate the antioxidant activity, a free radical scavenging assay was conducted using two free radical agents, 2,2-diphenyl-2-picrylhydrazyl (DPPH) and 2,2′-azino-bis(3-ethylbenzothiazoline-6-sulfonic acid (ABTS). As depicted in Figure 5, the SDW2-derived xanthophyll pigment exhibited 73.4 ± 1.4% DPPH and 84.9 ± 0.7% ABTS of radical scavenging activity at a dosage of 20 mg/L, with an IC_50_ value of 13.2 ± 0.4 mg/L for DPPH and 10.7 ± 0.2 mg/L for ABTS. The free radical scavenging activity of the SDW2-derived xanthophyll pigment was over 25.8% higher than that of deinoxanthin (DPPH (47.6 ± 1.7%), ABTS (54.7 ± 3.6%)), which had the strongest antioxidation activity among the xanthophylls tested in this study (Table 2). These results suggest that the yellow xanthophyll pigment produced by the *Erythrobacter* sp. SDW2 strain has great potential as a promising natural ingredient for industrial application.

## 3. Materials and Methods

### 3.1. Sample Collection and Isolation of Yellow-Pigmented Marine Bacterium

The sample was collected from coastal seawater on Jeju Island in the Republic of Korea (33°14′42.2″ N 126°24′41.7″ E). The collected seawater was serially diluted using 1.5% NaCl and spread onto a marine agar (MA) plate (KisanBio, Seoul, Korea). Culture plates were incubated at 37 °C until the colonies became visible. The yellow-pigmented single colony was purified by transferring it onto a new MA plate. The purely cultured, yellow-pigmented marine bacterial strain was suspended in 40% glycerol solution and stored at −80 °C. An isolate was routinely cultivated in MB at 37 °C.

### 3.2. Identification and Characterization of an Isolated Strain

Additionally, 16s rRNA gene sequencing was conducted to phylogenetically identify an isolate. To achieve this, genomic DNA was prepared from 3 mL of overnight liquid cell culture using the MEGAquick-Spin^TM^ Plus Total Fragment DNA Purification Kit (Intron, Korea) according to the manufacturer’s instructions. The 16s rRNA gene was amplified and directly sequenced using the universal primers 27F (5′-agagtttgatcmtggctcag-3′) and 1492R (5′-tacggytaccttgttacgactt-3′). The 16s rRNA sequence of the isolate was deposited into GenBank under the accession number MZ437366. The nearly complete sequence of the 16s rRNA gene was compared using the EzTaxon Server [28] and NCBI BLAST [29] to identify the members of the isolated bacteria. A phylogenetic tree was constructed based on the 16s rRNA genes of bacterial species similarly related to the isolate using the MEGA 7.0 program [30] with the neighbor-joining method [31] and Kimura two-parameter model [32] with bootstrap values based on 1000 replications. The identified 16s rRNA sequences were obtained from GenBank in the NCBI database.

The Gram staining and microscopic examination were performed as described previously [33]. The biological properties were characterized using the analytical profile indent (API)20NE strep kit (BioMerieux, Marcy l’Etoile, France) according to the manufacturer’s protocol. Catalase and oxidase activity were also evaluated using H_2_O_2_ and N, N, N^1^N^1^-tetramethyl-p-phenylenediamine-dihydrochloride (Sigma-Aldrich, St. Louis, MO, USA), respectively. Cell growth at different temperatures (20 to 45 °C), salinities (NaCl, 2% to 8%), and pH values (pH 4 to pH 9) was also monitored by measuring the optical density at 600 nm (OD_600_) using an Epoch microplate reader (Biotek, Winooski, VY, USA).

Whole-genome sequencing of an isolate was performed by Chunlab, Inc. (Seoul, Korea) using the Pacbio Sequel II platform [34]. Gene-finding and functional annotation pipeline of whole-genome assembles used the EzBioCloud genome database. Protein-coding sequences (CDSs) were predicted by Prodigal 2.6.2 [35]. Genome annotations related to carotenoid biosynthesis were validated using NCBI BLAST.

Cell density was monitored using an Epoch™ microplate reader (Biotek, Winooski, VY, USA) at a wavelength of 600 nm. The measured value was diluted with a culture medium to fall within the range of 0.2 to 0.8, and the final cell density was calculated by multiplying the measured value by the dilution factor. 

### 3.3. Xanthophyll Pigment Extraction and Analytical Methods

A fresh colony was inoculated in 3 mL of MB and cultivated overnight at 37 °C with agitation of 200 rpm to extract pigment from the isolate. The overnight cell cultures were transferred to 50 mL of MB in a 250 mL baffled shake flask and incubated for 48 h at 37 °C. After incubation, the cells were harvested at 4000 rpm for 30 min and washed twice using deionized water. The sample was freeze-dried and kept at −80 °C before pigment extraction. Crude xanthophyll pigment from 50 mg of lyophilized biomass was extracted using 10 mL of methanol (HPLC-grade, Daejung, Kiheung, Korea). All-trans fucoxanthinol (Merck, KGaA, Darmstadt, Germany) was used as the standard material to determine the xanthophyll pigment concentration produced by the SDW2 strain.

Xanthophyll pigment analysis was conducted using the previously described methods [18]. The UV–vis spectrum of the total xanthophyll extract was recorded from 300 to 700 nm using an Epoch microplate reader (Biotek, Winooski, VY, USA). The crude extracts were developed on a silica gel TLC plate (Merck, KGaA. Darmstadt, Germany) with an ethyl acetate/n-hexane solution (6:4, *v/v*) to separate the xanthophyll fraction. Moreover, the HPLC analysis was performed using Agilent 1260 Infinity II (Agilent Corp., Santa Clara, CA, USA) equipped with a Zorbax Eclipse XDB-C18 column (4.6×150 mm; Agilent Corp., Santa Clara, CA, USA) and a UV–vis detector at 450 nm. The injection volume was 5 μL, with a column temperature of 30 °C. The isopropanol, methanol, and acetonitrile (10:50:40, *v/v/v*) mixture was used as an eluent with 0.5 or 1 mL/min. The quantification of the residual carbon source in the liquid culture medium was determined using HPLC equipped with a refractive index detector. A MetaCarb 87H column (4.6 × 250 mm; Agilent Corp., Santa Clara, CA, USA) was used to separate carbons and organic acids.

### 3.4. Xanthophyll Pigment Production

Routinely, several fresh colonies were seeded in 3 mL of MB medium and cultivated overnight at 37 °C. Seed cultures (1%) were reinoculated in a 250 mL baffled flask with 50 mL of MB and cultivated at 37 °C and 200 rpm for 48 h. In addition, 5 g/L of glucose, fructose, glycerol, xylose, and sucrose were used, monitoring their biomass (OD_600_) and concentrations of carbon sources and xanthophylls to examine the effects of different carbon sources on cell growth and xanthophyll production. All experiments were conducted in triplicate, and the values were expressed as the mean ± the standard deviation.

### 3.5. Evaluation of Antioxidation Activity

The antioxidation activity of methanolic crude extracts was investigated based on 2,2-diphenyl-1-picrylhydrazyl (DPPH) and 2,2′-azino-bis(3-ethylbenzothiazoline-6-sulfonic acid) (ABTS) radical scavenging assays, as previously described [18,36], with some modifications. In brief, 0.2 mM DPPH (Alfa Aesar, Tewksbury, MA, USA) was dissolved in methanol. In addition, 100 μL of DPPH solution was reacted with 100 μL of xanthophyll pigment solution and incubated for 30 min in the dark. In the ABTS assay, the ABTS solution was prepared by mixing 2.45 mM of potassium persulfate (Tokyo Chemical Industry, Tokyo, Japan) and 7 mM of ABTS (Alfa Aesar, Tewksbury, MA, USA) and placed in the dark for 16 h. The absorbance of ABTS solution at 734 nm was adjusted to approximately 0.7 using methanol. Xanthophyll pigment solutions were diluted at different concentrations ranging from 1.25 to 20 mg/L, using methanol for the assays. Ascorbic acid (20 mg/L) was used as a positive control for each assay. The carotenoids zeaxanthin, lutein, fucoxanthin, fucoxanthinol, and astaxanthin were purchased from Sigma-Aldrich (St. Louis, MO, USA) and used to compare free radical scavenging activities. Deinoxanthin was obtained from the DX2 strain, as reported previously [18]. Antioxidation activity was calculated using the following equation:

Radical scavenging activity (%) = [(A_blank_ − A_sample_)/A_blank_] × 100, (1)

where A_blank_ is the absorbance of the control, and A_sample_ is the absorbance of the sample observed at 517 nm for DPPH and 734 nm for ABTS, respectively. The antioxidant activity of xanthophyll pigment was expressed as the IC_50_ values (the sample concentration required for inhibition of 50% of free radicals) for DPPH and ABTS. The values were expressed as the mean ± the standard deviation of at least three independent experiments.

## 4. Conclusions

In this study, we report, for the first time, the isolation and identification of pure xanthophyll pigment-producing marine bacterium *Erythrobacter* sp. strain SDW2. Through chromatographic profile and genome sequencing analysis, the yellow xanthophyll pigment produced by the SDW2 strain was predicted to be a zeaxanthin derivative. The SDW2 strain isolated in this study, unlike other marine microorganisms, can be a promising microbial host strain for industrial application due to its ability to produce homo-xanthophyll pigment, and its excellent performance in producing xanthophyll pigment. Furthermore, the yellow xanthophyll pigment derived from the SDW2 strain showed a strong antioxidant effect compared to other pigments. Additional research is underway to identify the exact structure of the yellow xanthophyll pigment derived from the SDW2 strain. Furthermore, the identification of its value as an industrial microorganism through process optimization and metabolic engineering will be the focus of our next study.

## Figures and Tables

**Table 1:** Biochemical characteristics of *Erythrobacter* sp. strain SDW2.

Test	Strain SDW2
Production of acid from glucose	−
Indole production	−
Nitrate reduction	+
Enzyme activity	
Catalase	+
Oxidase	+
Arginine dihydrolase	−
Urease	−
Beta-glucosidase	+
Beta-galactosidase	+
Protease	−
Assimilation	
D-glucose	+
L-arabinose	+
D-mannose	+
D-mannitol	+
*N*-acetyl-glucosamine	−
D-maltose	+
Potassium gluconate	+
Capric acid	−
Adipic acid	+
Maleic acid	+
Trisodium citrate	−
Phenylacetic acid	−

−, negative; +, positive.

**Table 2:** Comparison of free radical scavenging activity among xanthophylls (20 mg/L).

Carotenoids	Scavenging Activity (%)	
	DPPH	ABTS
Fucoxanthin	16.7 ± 4	32.1 ± 2.7
Fucoxanthinol	13.5 ± 2.7	24.3 ± 2.9
Lutein	23.7 ± 2.6	38.1 ± 0.7
Astaxanthin	32.5 ± 2.1	35.3 ± 2.8
Deinoxanthin	47.6 ± 1.7	54.7 ± 3.6
Xanthophyll pigment extracts (*Erythrobacter* sp. SDW2)	73.4 ± 1.4%	84.9 ± 0.7%
Ascorbic acid (positive control)	86.6 ± 4.9	92.9 ± 3.2

## Data Availability

Not applicable.
